# The Influence of Tool Pin Geometry and Speed on the Mechanical Properties of the Bobbin Tool Friction Stir Processed AA1050

**DOI:** 10.3390/ma15134684

**Published:** 2022-07-04

**Authors:** Mohamed M. Z. Ahmed, Mohamed M. El-Sayed Seleman, Rana G. Eid, Ibrahim Albaijan, Kamel Touileb

**Affiliations:** 1 Mechanical Engineering Department, College of Engineering at Al Kharj, Prince Sattam Bin Abdulaziz University, Al-Kharj 16273, Saudi Arabia; i.albaijan@psau.edu.sa (I.A.); k.touileb@psau.edu.sa (K.T.); 2 Department of Metallurgical and Materials Engineering, Faculty of Petroleum and Mining Engineering, Suez University, Suez 43512, Egypt; mohamed.elnagar@suezuniv.edu.eg (M.M.E.-S.S.); ranagamal1990@yahoo.com (R.G.E.); 3 Suez and Sinai Metallurgical and Materials Research Center of Scientific Excellence (SSMMR-CSE), Suez University, Suez 43512, Egypt; 4 Canal High Institute of Engineering and Technology, Suez 43512, Egypt

**Keywords:** AA1050, bobbin tool friction stir processing, mechanical properties, thermal cycle, torque, surface roughness, fracture surface

## Abstract

AA1050 plates of 8 mm thickness were processed via bobbin-tool friction stir processing technique at a constant rotation speed of 600 rpm and different travel speeds ranging from 50 to 300 mm/min using three-pin geometries of triangle, square, and cylindrical. The temperatures of the processed zone, the advancing side, and the retreating side were measured; the machine torque during processing was also recorded. The processed materials were evaluated in terms of surface roughness, macrostructure, tensile properties, and hardness measurements. The fracture surfaces of the tensile fractured specimens were investigated using SEM. The results indicated that the pin geometry and processing speed significantly affect the generated heat input and the morphology of the processed zone. The peak temperature in the center of the processed zone decreases with increasing the travel speed from 50 to 300 mm/min at all applied pin geometries. The maximum temperature of ~400 °C was reached using the cylindrical pin geometry. The machine torque increases with increasing the travel speed at all applied pin geometries, and the highest torque value of 73 N.m is recorded using the square pin geometry at 300 mm/min travel speed. The top surface roughness of the processed area using the cylindrical pin is lower than that given by the other pin geometries. Under all applied conditions, the hardness of the processed area increases with increasing travel speed, and the cylindrical pin shows a higher hardness than the other pin geometries with 19% enhancement over the BM. The AA1050 processed using a cylindrical pin at 200 mm/min travel speed and a rotation speed of 600 rpm produces a sound processing zone with the highest ultimate tensile strength of 79 MPa.

## 1. Introduction

AA1050 alloy has received particular attention in many industrial sectors due to its exceptional properties: low density, formability, ease of fabrication, excellent ductility, high electrical conductivity, and high corrosion resistance [1,2]. Based on friction stir welding (FSW) principles, friction stir processing (FSP) has been developed as a solid-state technique for processing ferrous [3,4,5] and nonferrous alloys [6,7,8,9]. In recent years, FSP technology using a conventional tool (CT) design has been widely applied to aluminum and its alloys only to modify the microstructure of the material surface with and without ceramic particles additions [10,11]. For AA1050 alloy, many researchers have paid attention to utilizing the FSP technique with a CT design for welding [12,13,14] and processing [15,16,17] based on different parameters. Bobbin tool (BT) design is an innovative tool design and was initially used in the FSW of aluminum alloys [18,19,20,21]. The BT refers to the tool shape, two shoulders (lower and upper) connected by a pin. The bobbin tool friction stir welding (BT-FSW) has several advantages over the FSW using CT. The two shoulders offer higher symmetric frictional heat contribution through the welded full-thickness compared to the CT-FSW, resulting in more uniform plastic deformation in the stir zone (SZ) and lower distortion of the produced welded joints [22]. Moreover, the lower shoulder of the BT-FSW has another benefit, as it is used instead of the supporting backing plate with the CT-FSW; thus, the BT-FSW of hollow structures is utilized [19,23]. Thus, many researchers used the BT design to weld aluminum alloys due to its many advantages in producing high-quality welds. Li et al. [24] studied the effect of BT rotation speeds of 600–1200 rpm at a constant travel speed of 500 mm/min using a cylindrical (Cy) pin. They found that the maximum tensile strength (262.7) was obtained at a rotation speed of 800 rpm. The effect of travel speeds ranging from 130 to 250 at 350 rpm rotation speed on the BT-FSW of AA2219 was investigated by Wen et al. [25]. They reported that the grain size was decreased with increasing the welding speed. The maximum tensile strength (330 MPa) with 70% joint efficiency was observed at 210 mm/min travel speed. Fuse and Badheka [26] investigated the effects of BT shoulder diameters (20, 22 and 24 mm) using 8 mm Cy pin diameter on the BT-FSW of AA6061-T6 alloy at a constant rotation speed of 380 rpm and 31.5 mm/min. They reported that the best-welded joint in terms of tensile properties was produced using the 24 mm shoulder diameter. Liu et al. [27] studied the effect of two different BT pin geometries (threaded and threaded with three planes) on the welding of 5A05-H112 at a constant rotation speed of 250 rpm and 200 mm/min travel speed. They remarked that the macro of dumbbell-shaped and drum-shaped appearances correspond with the two-pin geometries. BT-FSW of dissimilar AA2219 and AA6056 alloys was studied by Wen et al. [28]. They concluded that an excellent mixed material was observed at the bonding interface. It can be seen from the above survey and available literature that BT has been intensively used in welding different aluminum alloys. This gives great motivation to the researchers to apply the BT in the processing of aluminum alloys to modify the microstructure or produce composites. There is a lack of studies on the BT-FSP of aluminum and aluminum alloys. In addition, until now, one research utilized the BT-FSP to produce AA6061/B_4_C composite [29]. They only studied the effect of the number of paths on AA6061/B_4_C composite properties processed at a constant 350 rpm and 31.5 mm/min. The produced composites using three paths displayed a more uniform dispersion of B_4_C particles with 25% hardness enhancement over the base material (BM). In fact, the quality of the processed material using the BT-FSP technique is related to many parameters such as pin geometries, processing travel speeds, rotational speeds, and shoulder features. These factors affect the internal material flow of the SZ, the heat generation, and then the properties of the processed materials. Therefore, this work aims to determine the influence of BT pin geometries (cylindrical, triangle, and square) and the processing travel speeds (50, 100, 200, and 300 mm/min) at a constant rotation speed of 600 rpm on the mechanical properties of the processed 8 mm thickness AA1050 in terms of hardness and ultimate tensile strength. Moreover, the BT-FSP thermal cycle including processing zone, advancing side, and retreating side temperatures in light of the applied torque was measured and discussed. 

## 2. Materials and Methods

AA1050 plates with dimensions of 150 mm length, 75 mm width, and 8 mm thick, were supplied by Egyptian Aluminum Company, Egypt, and processed using the BT-FSP technique. The chemical composition of the as-received AA1050 was investigated using Foundry-Master pro, (Oxford Instruments, Abingdon, UK). The chemical composition and mechanical properties of AA1050 alloy are listed in Table 1.

**Figure 1:**
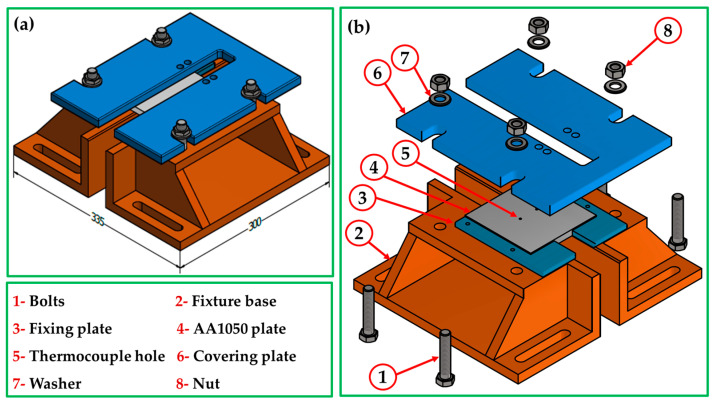
BT-FSP fixture setup configuration to process AA1050. (**a**) Assembled view (**b**) Exploded view.

**Figure 2:**
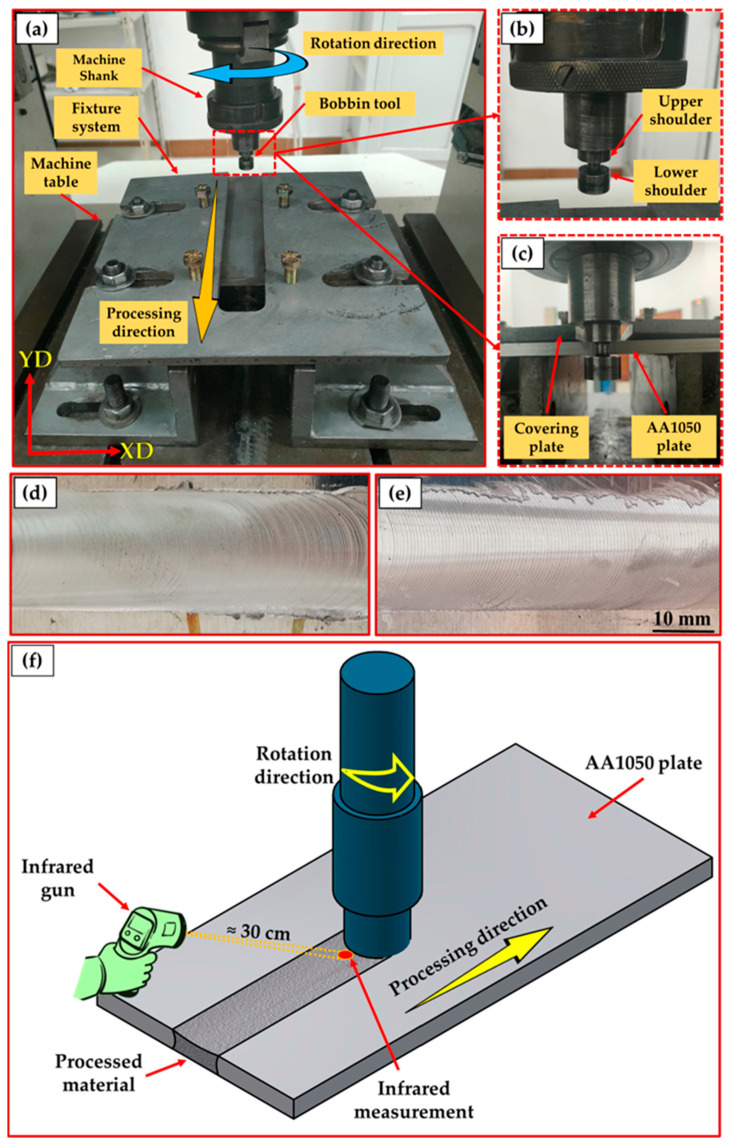
(**a**,**b**) Front images of the BT-FSP setup configuration, (**c**) back view of the BT location before processing, and example of (**d**) the upper and (**e**) lower surface of a processed specimen. (**f**) A schematic showing measuring temperature in the PZ using an infrared thermometer.

For BT-FSP of AA1050 experiments, the plates were first fixed on the machine table using a fixture designed for BT-FSP purposes (Figure 1), and the BT was fixed in the machine shank, as shown in Figure 2a. Then BT-FSP of AA1050 was carried out using the EG-1 FSW/FSP machine (Suez University, Suez, Egypt) [30]. The experimental setup of BT-FSP of AA1050 is shown in Figure 2a–c, and an example of upper and lower surface for the AA1050 BT-FSPed materials is shown in Figure 2d,e.

**Figure 3:**
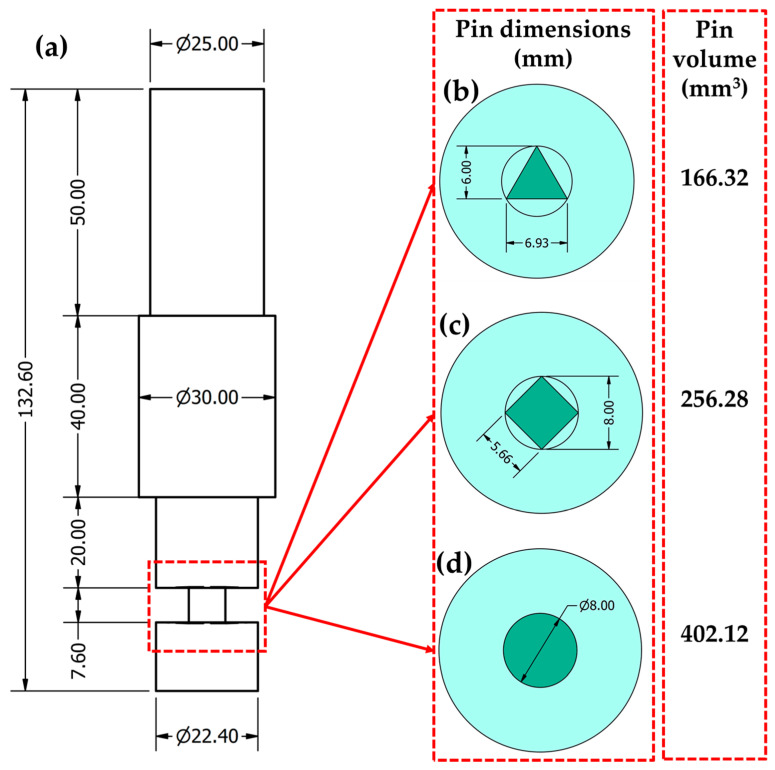
(**a**) Dimensions and volumes of the BT (all dimensions in mm) and the BT pin profiles; (**b**) Tr, (**c**) Sq, and (**d**) Cy.

The influence of processing travel speed and pin geometry on BT-FSP of AA1050 was investigated using different travel speeds of 50, 100, 200, and 300 mm/min and different pin geometries of triangular (Tr), cylindrical (Cy), and square (Sq) at a constant rotation speed of 600 rpm. The plunge depth of both the upper and lower tool shoulders was constant and set to be 0.2 mm. The dimensions of the top and the bottom shoulders have the same diameter of 25 mm with three grooves. The diameter of the Cy pin is 8 mm. Moreover, the Tr and Sq pins were formed inside the circle having an 8 mm diameter. The volume for each tool pin has been calculated and the values were 166.32, 256.28, and 402.12 mm^3^ for the Tr, Sq, and Cy, respectively. Figure 3 represents (a) the main dimensions and volumes of the BT and pin profiles of (b) Tr, (c) Sq, and (d) Cy. The BT-FSP tools were machined from W302 cold worked tool steel and were heat-treated to get a 52 HRC. 

**Figure 4:**
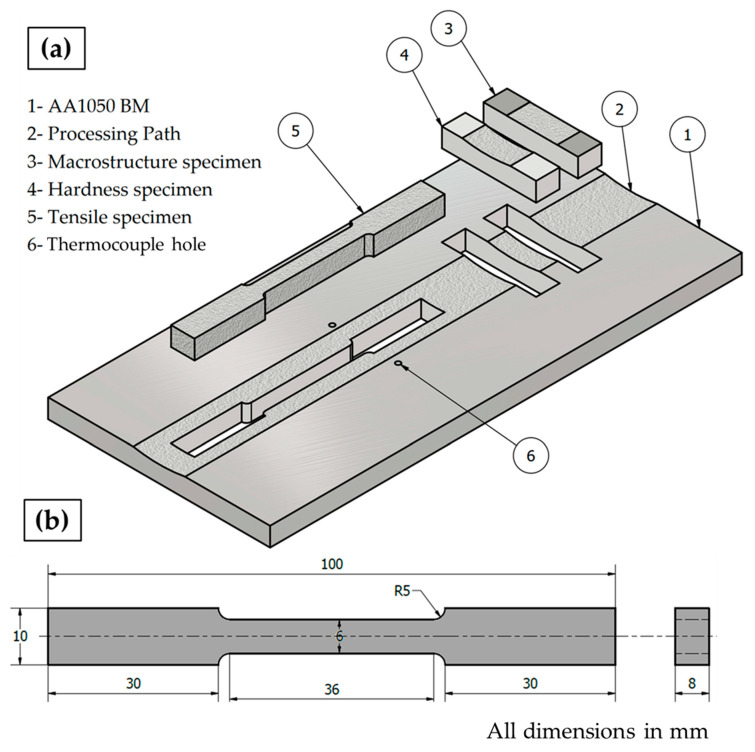
Schematic drawing shows (**a**) the location of cutting test specimens for the processed materials and (**b**) the dimensions of tensile test specimens (thermocouple in Figure 4).

The BT-FSP of AA1050 temperature in the processed zone (PZ) during the BT-FSP was measured and recorded using an infrared thermometer (Quicktemp 860-T3, Testo Company—Berlin, Germany) for all the processed samples. The temperature was measured behind the tool at a constant distance of 30 cm (Figure 2f). Moreover, the temperatures on the advancing side (AS) and the retreating side (RS) were measured and recorded by the Modern Digital Multimeter (MDM) model (UT61B-Zhejiang, China) using two thermocouples (type K). The locations of these thermocouples are given in Figure 4a. The surface roughness of the processing paths for all processed materials was measured using PosiTector Surface Profile Gages (Ogdensburg, NY, USA). The PZ areas achieved using the different pin geometries were measured using the AutoCAD mechanical (Student version) software 2022. The bobbin tool friction stir processed (BT-FSPed) AA1050 materials and the AA1050 base material were cut perpendicular to the processing path direction (Figure 4a) for macrostructure and hardness examinations. The tensile test specimen was cut parallel to the processing path direction to evaluate the AA1050 processed materials as shown in Figure 4a,b, representing the dimensions of the tensile test samples according to ASTM-E8. The processed cross-section specimens for macrostructure investigation were ground with different emery paper up to 2400 and polished using an 0.05 µm Al_2_O_3_ paste, followed by etching with Keller’s reagent. The macrostructure examinations were carried out using Stereomicroscope Model: Optika SZR-10, OPTIKA, Ponteranica (BG)-Italy. The tensile properties in terms of the ultimate tensile strength (UTS) and elongation percentage (E%) of the processed materials and BM were evaluated using a universal testing machine (model: WDW-300D Testing Machine, 30-ton, Guangdong, China) at room temperature. Four samples at each BT-FSPed condition were used for tensile testing. The fracture surfaces of the tensile failed specimens were examined using a scanning electron microscope (SEM, Thermo-Scientific, Quattro S, Waltham, MA, USA).

**Figure 5:**
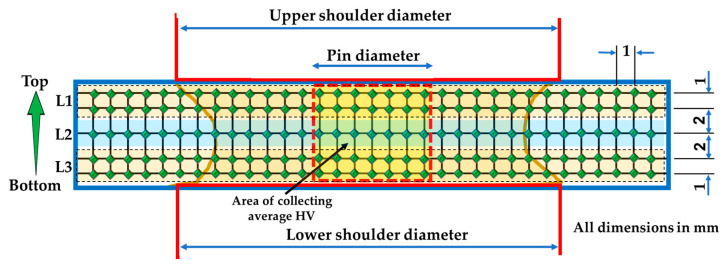
Schematic drawing of the indentation locations to perform hardness measurements.

To evaluate the hardness of the BT-FSPed AA1050, the cross-section of the processed materials is divided into five lines to present three layers: upper layer (L1), middle layer (L2), and lower layer (L3), as shown in Figure 5. Hardness were measured at all layers on the polished cross-sections by a hardness tester machine (HWDV-75, TTS Unlimited, Osaka, Japan) with a load of 0.3 kg and a dwell time of 15 s. The measurements were repeated for three specimens at each condition and plotted in terms of hardness contour map and average hardness.

## 3. Results and Discussion

### 3.1. BT-FSP Temperature

**Figure 6:**
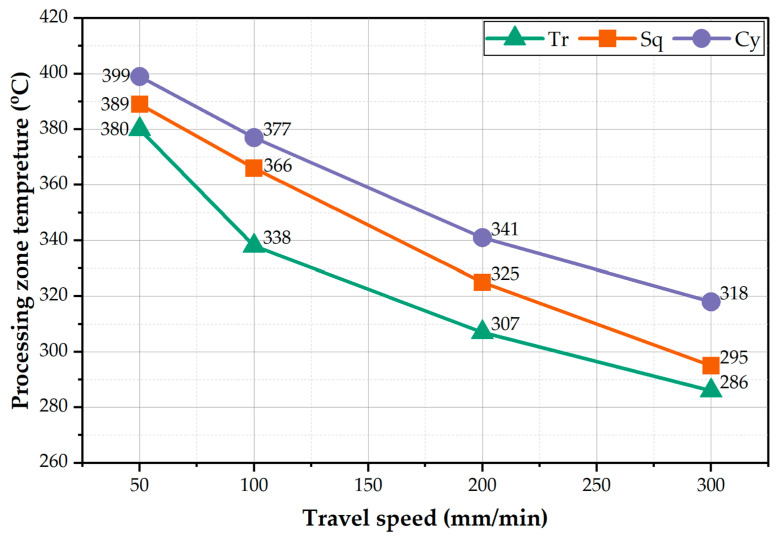
PZ temperature against travel speed at the used different pin geometries.

The processed zone (PZ) temperature was measured during the BT-FSP of 8 mm thickness AA1050, and the average values were plotted versus the different processing travel speeds of 50, 100, 200, and 300 mm/min for the different pin geometries of Tr, Sq, and Cy, as illustrated in Figure 6. It can be noted that the temperature of PZ decreases as the processing travel speed increases for all the used pin geometries. In addition, among all the BT-FSP, the Cy pin geometry shows the highest PZ temperatures compared to Tr and Sq pins at all processing travel speeds. In contrast, the Tr pin geometry provides the lowest PZ temperatures at the applied range of processing travel speeds. By applying the lowest processing travel speed of 50 mm/min, the highest PZ temperatures of 380, 389, and 399 °C are obtained using the pin geometries of Tr, Sq, and Cy, respectively. Whereas with applying the highest processing travel speed of 300 mm/min, the lowest PZ temperatures of 286, 309, and 318 °C are attained using the Tr, Sq, and Cy pins, respectively. For BT-FSP using the Tr pin, the stirring volume in the PZ is minimum due to the small pin volume relative to the other tool pins. Since a large volume pin must generate more frictional heat during the BT-FSP, thus, the frictional heat generated by the Tr pin should be lower than that given by the other pin geometries. The frictional heat generated by the Cy pin showed the highest PZ temperature values. Moreover, as the processing travel speed increases, the heat input per unit length along with the processing path decreases, and heat dissipation increases resulting in lower stir zone temperature compared to in case of decreasing the processing travel speed.

**Figure 7:**
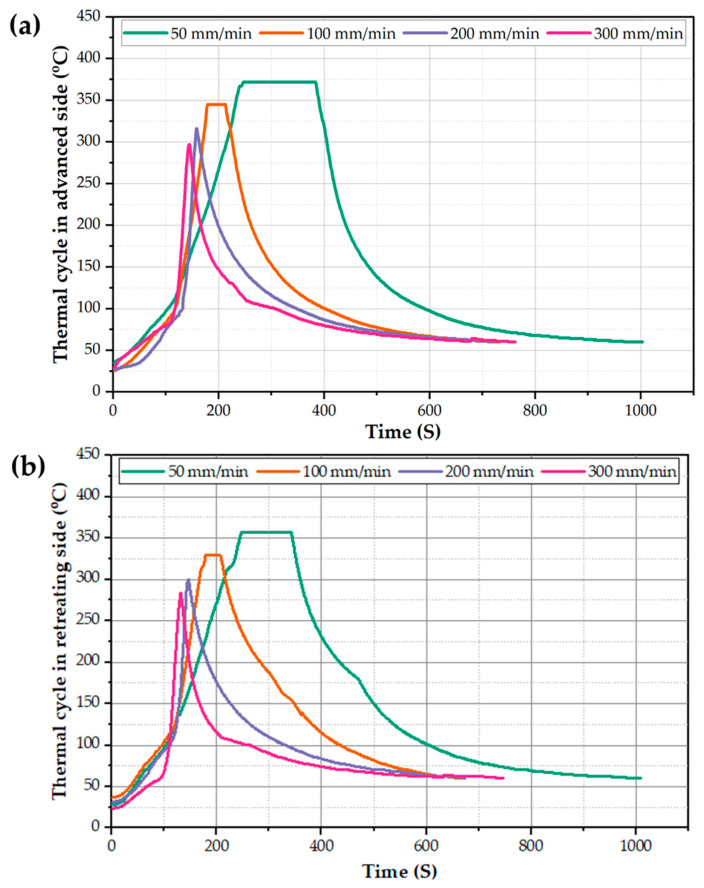
The thermal cycles of the (**a**) AS and (**b**) RS against time for the AA1050 BT-FSPed at a constant rotation speed of 600 rpm and different travel speeds of 50, 100, 200, and 300 mm/min using Cy pin geometry.

The study of thermal cycle history is essential for the analysis of the material flow and mixing during the BT-FSP. Thus, the thermal cycle at AS and RS as a function of time for the BT-FSPed AA1050 at a constant rotation speed of 600 rpm and the different processing travel speeds of 50, 100, 200, and 300 m/min using the Cy pin were recorded and presented in Figure 7a,b, respectively. It can be remarked that the BT-FSP thermal cycle of both AS and RS is divided into three stages. First, insert the rotating BT tool at the applied rotation speed of 600 rpm into the suggested processing zone centerline of the fixed 8 mm AA1050 plate at a slow processing travel speed of 20 mm/min to pre-heat the AA1050 material. Second, apply a 20 s holding time to attain enough temperature for stirring action during BT-FSP. The measured temperature of the first and second stages was around 110 °C. Third, applying the required processing travel speed to start conducting the processing pass of AA1050, in this stage, the temperature rises gradually during BT-FSP to reach the peak temperature. After ending the BT-FSP, the temperature of the processed specimens gradually decreases during the air cooling. The RS shows a peak temperature of 357 °C, and the AS shows a peak temperature of 372 °C using Cy pin at a travel speed of 50 mm/min. It should be mentioned that the same trend of thermal cycles was obtained using the other pin geometries (Tr and Sq) at the currently applied processing speed parameters with a difference in the recorded peak temperatures of the thermal cycle at each processing travel speed.

**Figure 8:**
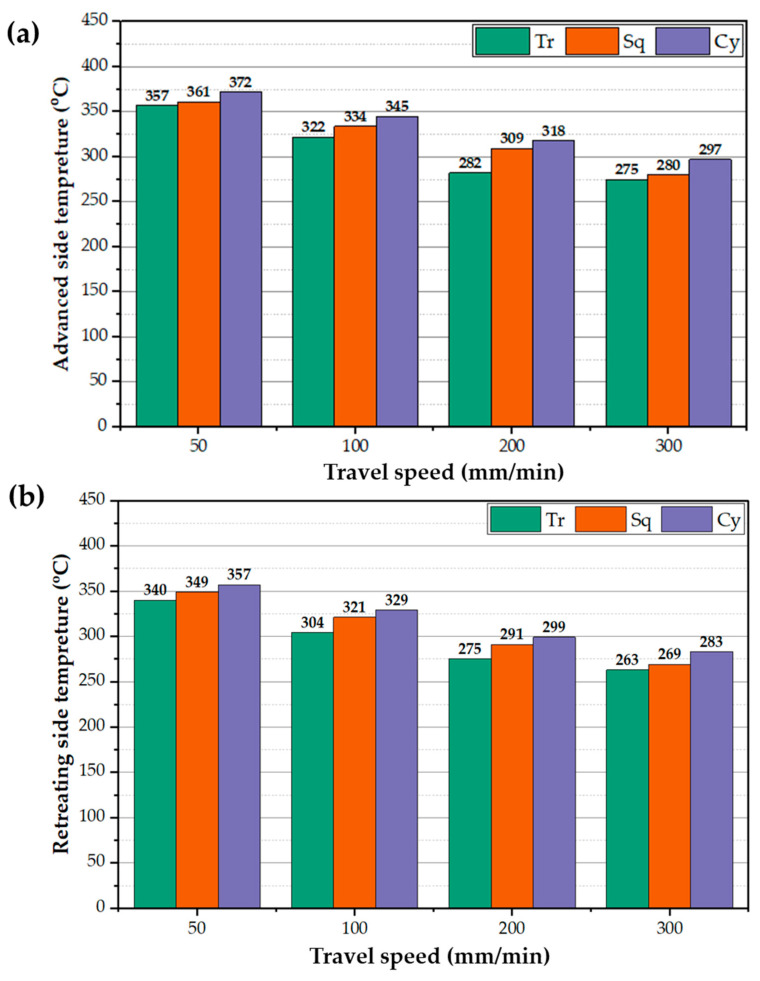
Peak temperatures of (**a**) AS and (**b**) RS for the BT-FSPed AA1050 at a constant rotation speed of 600 rpm and different travel speeds of 50, 100, 200, and 300 mm/min using Tr, Sq, and Cy pin geometries.

Figure 8a,b displays the recorded peak temperatures during BT-FSP of AA1050 using the different pin geometries at different processing speeds at the AS and RS, respectively. In general, the temperature of the AS and RS decreases with increasing processing travel speed. And the BT with Cy pin generates higher temperatures on both sides than the other pin geometries. In contrast, the Tr pin generates lower temperatures on both sides. The recorded temperatures of the AS are slightly higher than the RS at all the pin geometries. After the BT penetration, the plasticized materials flow around the pin and transfer from the AS to RS during BT-FSP. The transferred materials cooled in the RS [31]. The AS generates higher shear stress (friction force) than the RS during stirring action and generates more frictional heat [32,33,34].

### 3.2. BT-FSP Torque

**Figure 9:**
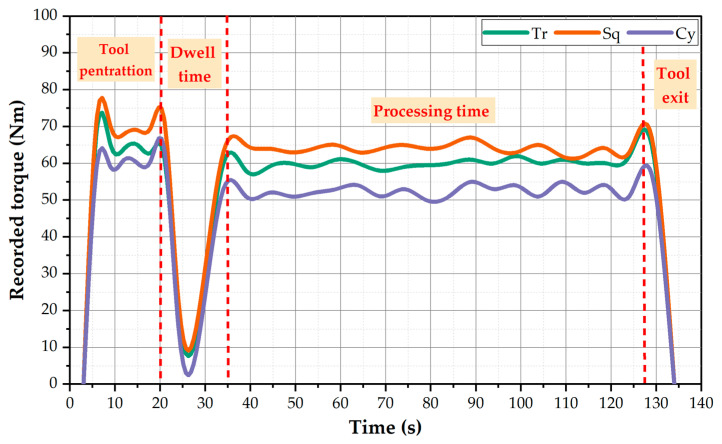
The recorded torque against the BT-FSP time at 600 rpm and 100 mm/min using different pin geometries to process 8 mm AA1050.

The BT-FSW and BT-FSP are related to many factors affecting the success of the process. Among these factors, torque is considered one of the essential parameters to achieving high-quality joint and processed materials. The heat input generated by the stirring action between the tool and the material under processing depends on the applied torque. Thereto, monitoring and governing the BT-FSP torque is vital for expecting the BT life and performance. The value of BT-FSP torque presented in the monitor of the FSW and FSP full-automatic machine in the current study can be used as an indicator for the material’s resistance to moving around the pin during the BT-FSP. The recorded torque during BT-FSP of AA1050 using different pin geometries (Tr, Sq, and Cy) at the processing travel speed of 100 mm/min and 600 rpm rotational speed are shown in Figure 9. The plotted torque data reveals four regions: (1) tool penetration, (2) dwell time, (3) material processing, and (4) tool exit. These distinct regions are typically similar to that reported by Ahmed et al. [19] to BT-FSW AA1050 lap joints. In the beginning, the BT moves to penetrate the workpiece during tool penetration, and the torque value sharply increases to attain the maximum value for all pin geometries. After that, 20 s was applied as a dwell time to achieve pre-heating. In this region, the torque values decrease to the minimum value (around 10 N.m) due to a low amount of stirring material around the BT. In the third region (processing time), the torque values rise again because the material around the tool resists the stirring process in the PZ. In this stage, the processed path is achieved with nearly a steady-state torque value. The recorded values of BT-FSP torque are around 63, 60, and 52 N.m using BT with pin geometries of Sq, Cy, and Tr, respectively. Finally, the torque curve decreases sharply due to the BT exiting from the workpiece (process end).

**Figure 10:**
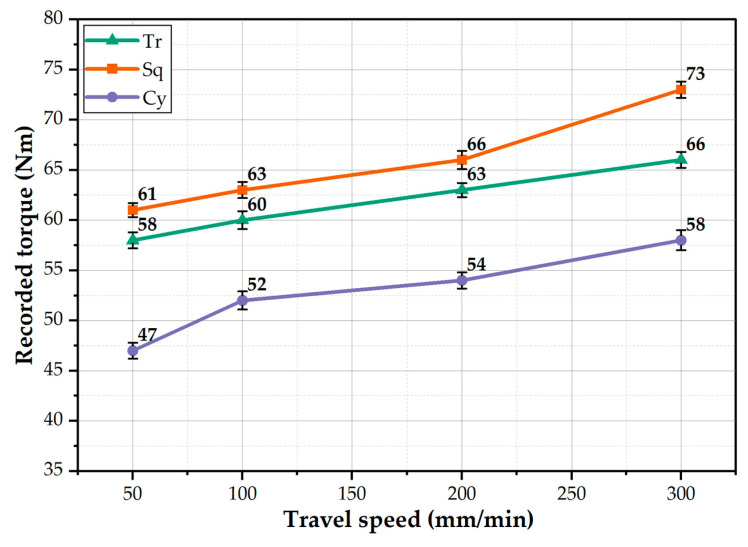
The average recorded torque as a function of processing travel speed at different pin geometries.

The average torque values during BT-FSP of AA1050 were calculated with the applied pin geometries at the range of the processing travel speed and presented in Figure 10. It can be seen that the measured torque values for all pin geometries increase with the increase in travel speed from 50 to 300 mm/min; with increasing the travel speed, the heat input decreases and leads to difficulty in moving the processed material around the tool, also hindering the tool from traveling through the material. Furthermore, it can be mentioned that the average torque value increases with increasing the pin volume. It can be recommended that the torque required with the Cy pin geometry (large volume) to achieve the AA1050 processing path is lower than that needed by the other pin geometries, Tr (small volume) and the Sq (intermediate volume) pins. This result should be considered in designing the tool pin.

### 3.3. Surface Roughness and Macrostructure Evaluation

**Figure 11:**
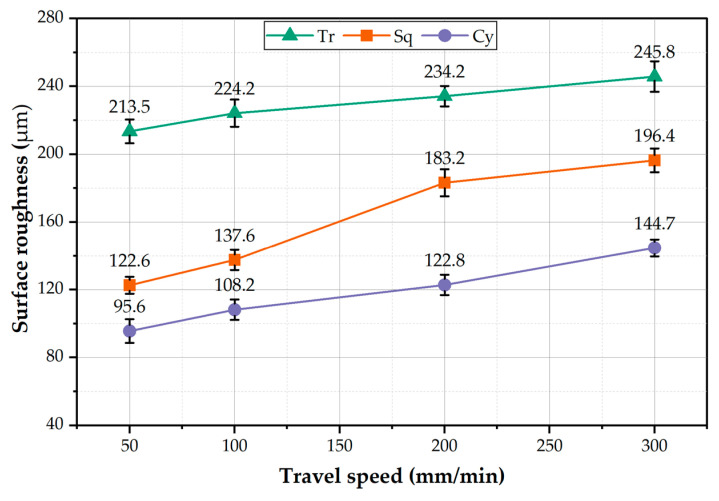
The surface roughness of the BT-FSPed AA1050 as a function of the travel speed for the used pin geometries.

**Figure 12:**
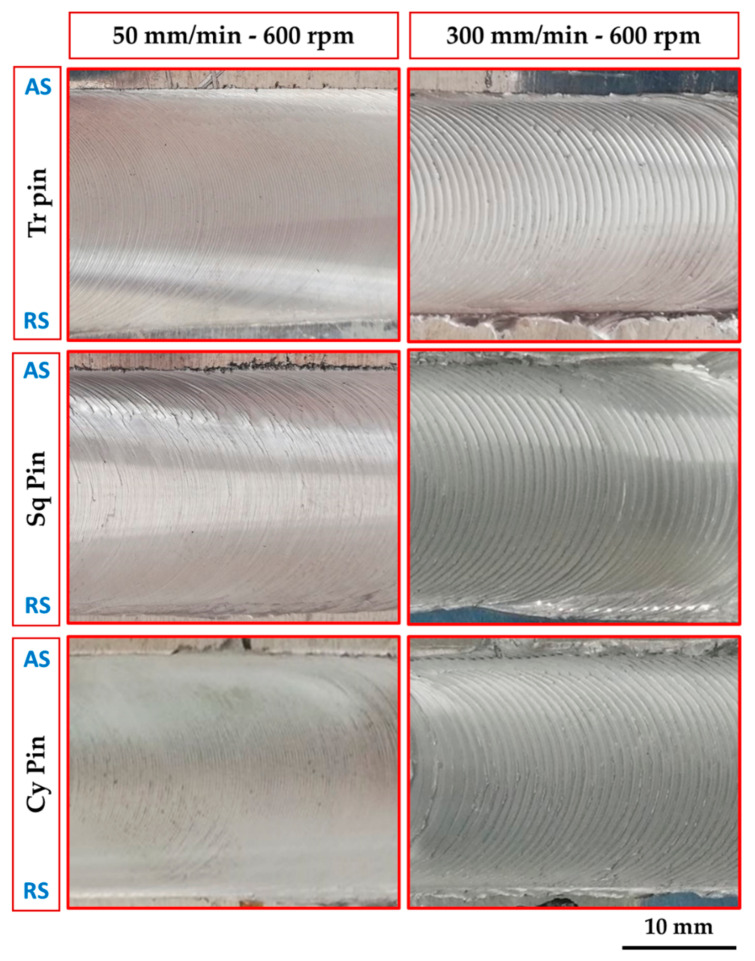
Photo images showing the macro-morphology of the BT-FSPed AA1050 at 50 and 300 travel speeds and a constant rotation speed of 600 rpm using different pin geometries.

The surface roughness or finish of a component under loading is considered one of the important parameters in determining its performance and lifespan. The surface roughness of the BT-FSPed AA1050 specimens at different travel speeds of 50, 100, 200, and 300 mm/min and a constant rotation speed of 600 rpm using Tr, Sq, and Cy pin geometries was measured and plotted in Figure 11. It can be seen that for all the used pin geometries, the average roughness value increases with increasing the processing travel speed. Among the used pin geometries, the Tr pin shows the highest surface roughness values compared to those with the Sq and Cy pin geometries. Using the Tr pin geometry attains the highest surface roughness value of 245.8 µm at 300 mm/min travel speed. In contrast, the lower surface roughness values of 95.6 µm attain at 50 mm/min using the Cy pin. The height of the ripples and the distances between them express the surface roughness of the processed materials. Figure 12 represents the photo images showing the macro-morphology of the BT-FSPed AA1050 at 50 and 300 travel speeds and a constant rotation speed of 600 rpm using different pin geometries. It can be noted that the height and the distance between the ripples increase with increasing travel speed. For all the used pin geometries, the height and distance between the ripples (the surface roughness) at 50 mm/min travel speed are lower than that given at 300 mm/min travel speed, as shown in Figure 12. The appearance of the weld and/or the processed surface that is formed beneath the tool shoulder after the FSW and FSP is directly related to the heat input in terms of the stirring process parameters and its main features are represented by the distance between ripples. The distance between the ripples is governed mainly by the rotation speed [24,35] and the travel speed [35]. Li et al. remarked that the distance between the ripples decreases with increasing the rotation speed from 600 to 1200 rpm at a constant travel speed of 500 mm/min for the BT-FSWed 6082-T6 Aluminum alloy. They detected the highest surface roughness at the welding condition of 1200 rpm and 500 mm/min. Shigematsu et al. [35] Studied the effect of the rotation speeds and the travel speed on the surface roughness of the dissimilar friction stir welding of A5052P-O aluminum and AZ31B-O magnesium alloys. They reported that the surface roughness of the SZ decreases with increasing the tool rotation speed from 1000 to 1400 rpm at a constant travel speed of 300 mm/min. Low surface roughness is attained at a rotation speed of 1400 rpm and a travel speed of 300 mm/min. They also reported that the increase in the travel speed led to the decrease in the surface roughness of the SZ at the welding condition from 100 to 500 mm/min travel speeds and a constant rotation speed of 1400 rpm. The low surface roughness attains at the travel speed of 500 mm/min and the rotation speed of 1400 rpm.

**Figure 13:**
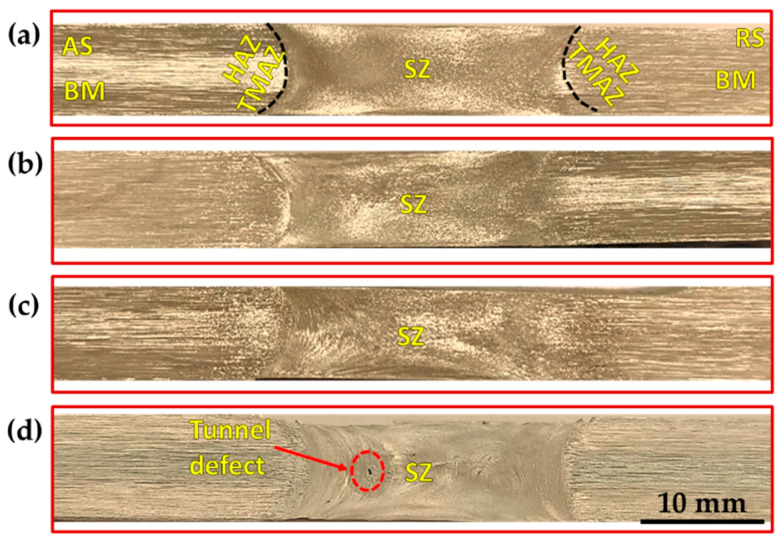
Macrostructure of the AA1050 BT-FSPed specimens at a constant rotation rate of 600 rpm using Tr pin (where BM: base material, SZ: stir zone, HAZ: heat-affected zone, and TMAZ: thermo-mechanically affected zone) and different travel speeds of (**a**) 50 mm/min; (**b**) 100 mm/min; (**c**) 200 mm/min, and (**d**) 300 mm/min.

**Figure 14:**
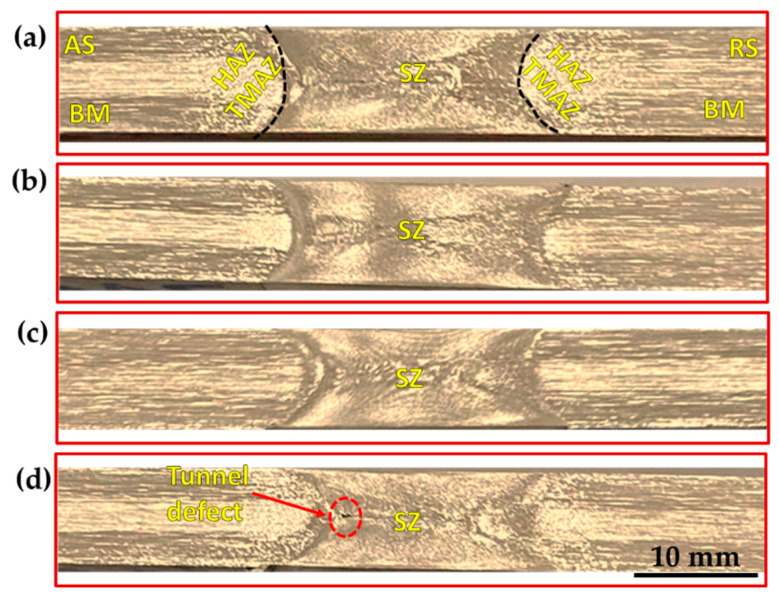
Macrostructure of the AA1050 BT-FSPed specimens at at a constant rotation rate of 600 rpm using Sq pin (where BM: base material, SZ: stir zone, HAZ: heat-affected zone, and TMAZ: thermo-mechanically affected zone) and different travel speeds of (**a**) 50 mm/min; (**b**)100 mm/min; (**c**) 200 mm/min, and (**d**) 300 mm/min.

**Figure 15:**
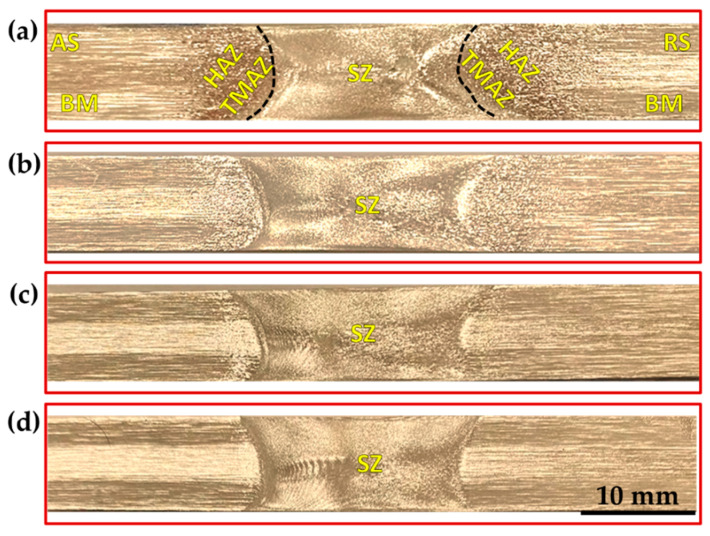
Macrostructure of the AA1050 BT-FSPed specimens at a constant rotation rate of 600 rpm using Cy pin (where BM: base material, SZ: stir zone, HAZ: heat-affected zone, and TMAZ: thermo-mechanically affected zone) and different travel speeds of (**a**) 50 mm/min; (**b**) 100 mm/min; (**c**) 200 mm/min, and (**d**) 300 mm/min.

Figure 13, Figure 14 and Figure 15 show the macrostructures of the AA1050 BT-FSPed at travel speeds of 50, 100, 200, and 300 mm/min and a constant rotation speed of 600 rpm using three-pin geometries of Tr, Sq, and Cy, respectively. The typical regions of SZ, thermo- mechanically affected zone (TMAZ), heat-affected zone (HAZ), and BM can be observed at all the applied processing parameters. It can be seen also that a sharp transition between the SZ and the BM appears on the AS, while a more diffuse transient region is obtained in the RS on the cross-section of the processed materials. This is ascribed to the different behaviors of the material flow on both sides (AS and RS) during the BT-FSP. Furthermore, the macrostructure of the AA1050 BT-FSPed using Cy pin geometries reveals sound processing zones at all applied travel speeds from 50 to 300 mm/min, as shown in Figure 15. Tiny tunnel defects were only observed on the cross-sections of the processed material produced at 300 mm/min travel speed for the processed material using the Tr (Figure 13d) and Sq (Figure 14d) pin geometries. Due to improper stirring, these tunnels are expected with a lower heat input (lower PZ temperature, Figure 6). Many works [23,36] showed that inadequate movement around the pin during the stirring process and insufficient heat input in the SZ lead to various defects such as cavities, kissing bonds, and tunnel defects.

**Figure 16:**
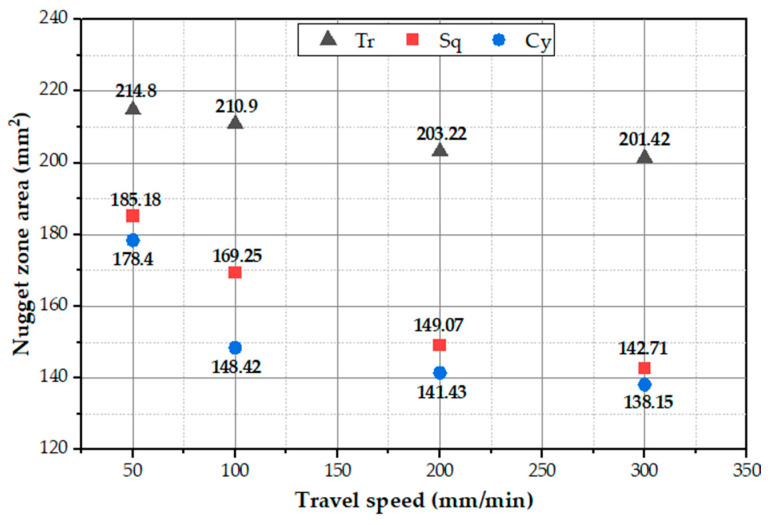
Processing zone area of the AA1050 BT-FSPed at travel speeds of 50, 100, 200, and 300 mm/min using Tr, Sq, and Cy pin geometries.

The PZ areas for all the applied processing conditions are measured and plotted in Figure 16. It can be seen that the PZ areas decrease with the increase in travel speed. In fact, the processing zone area is governed by the amount of heat input introduced to the PZ. The higher the heat input, the higher the material plasticity and the higher the PZ area. The heat input increases with decreasing the travel speed. Thus, the maximum PZ areas of 214.80, 185.18, and 178.40 mm^2^ are observed at a 50 mm/min lower travel speed using the Tr, Sq, and Cy pin geometries, respectively. In contrast, the minimum PZ areas of 201.42, 142.71, and 138.15 mm^2^ of AA1050 BT-FSPed at a higher travel speed of 300 are obtained using Tr, Sq, and Cy pin geometries, respectively.

### 3.4. Mechanical Properties

**Figure 17:**
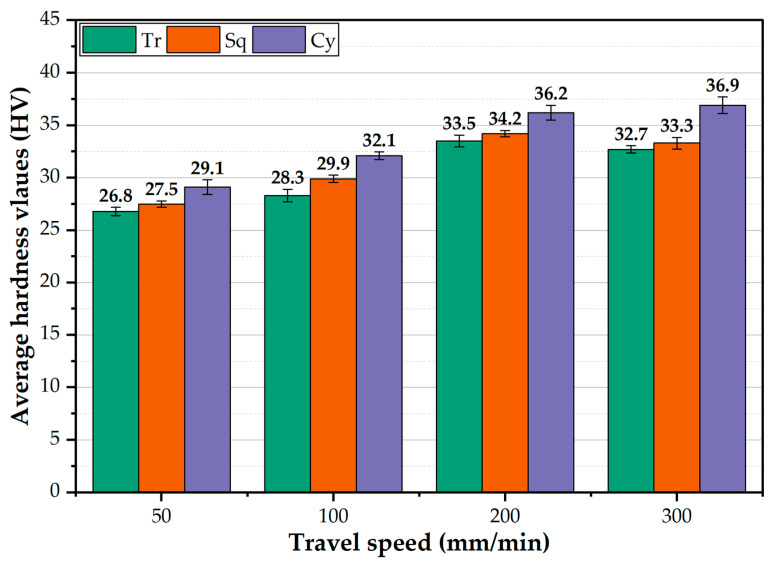
The AA1050 PZ average hardness values using the Tr, Sq, and Cy pin geometries at the applied travel speeds from 50 to 300 mm/min and a constant rotation speed of 600 rpm.

**Figure 18:**
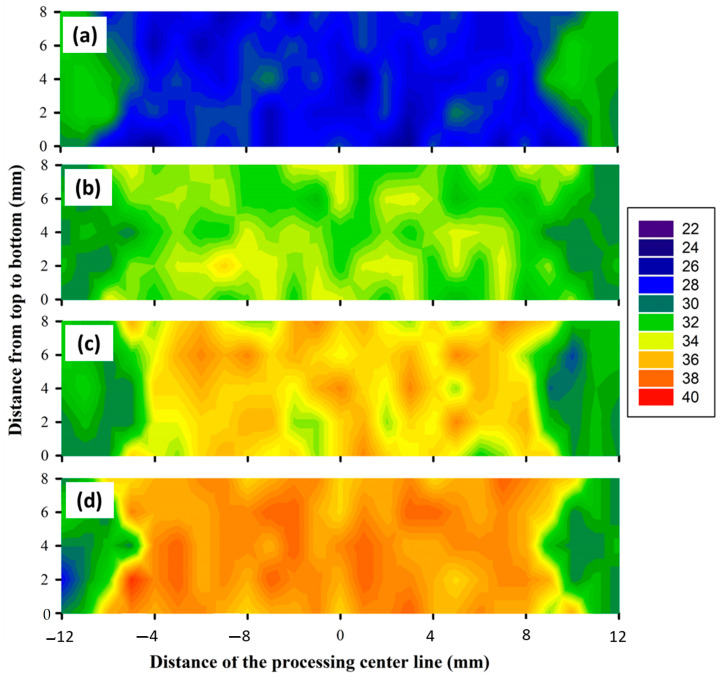
Hardness contour maps for the AA1050 BT-FSPed using Cy pin geometry at (**a**) 50, (**b**) 100, (**c**) 200, and (**d**) 300 mm/min, respectively.

Hardness is considered one of the essential mechanical properties and indicators of the microstructure change associated with friction stir welding and processing. Thus, the average hardness values for all the BT-FSPed AA1050 materials at the applied processing conditions were measured and analyzed. Moreover, the hardness contour maps across the BT-FSPed using the Cy pin geometry were plotted as an example to evaluate the hardness values through the 8 mm thickness of AA1050. Figure 17 shows the PZ average hardness values of the AA1050 BT-FSPed using the Tr, Sq, and Cy pin geometries at 50, 100, 200, and 300 mm/min travel speed and a constant rotation speed of 600 rpm. Generally, the hardness values increased with increasing the travel speed from 50 to 300 mm/min using all the pin geometries. This hardness trend is likely due to the decrease in heat input with increasing travel speed [20,37,38]. The decrease in the heat input will result in colder plastic deformation conditions which suppress the grain coarsening upon dynamic recrystallization and result in a significant grain size reduction in the processed zone. This behavior has been reported by Ahmed et al. [39] for friction stir welded AA7075 and AA5083, where a significant grain refining occurred in the nugget (NG) zone of AA7075 with an average grain size of 6 μm at 50 mm/min welding speed that was reduced to 2 μm by increasing the welding speed to 200 mm/min, and in case of AA5083 joints, NG zone the average grain size of 9 μm at 50 mm/min was reduced to 3 μm at 200 mm/min. This reduction in the grain size will cause the hardness to increase in the processed zone. A lower hardness was obtained using the Tr pin geometry than the other pin geometries, where the hardness in the PZ attains 86.4, 91.2, 101.1, and 101.0% of the AA1050 BM hardness (Table 1), while using Sq pin, the hardness values achieved 93.8, 101.3, 117.0, and 119.0% at the travel speed of 50, 100, 200, and 300 mm/min, respectively. The hardness values of the processed specimens using Sq pin geometry fall in between the values reached using the other pin geometries. Figure 18a–d represents the Vickers hardness contour maps of AA1050 BT-FSPed at the travel speeds of 50, 100, 200, and 300 mm/min and a constant rotation speed of 600 rpm using Cy pin geometry. For the applied travel speeds, it can be seen that a significant increase in the hardness can be observed in the hardness map due to the increase in the processing travel speed. The hardness is mainly affected by the generated thermal cycle experienced during FSP. As noted above from the temperature measurements, the increase in the processing travel speed from 50 to 300 mm/min has resulted in a significant reduction in temperature and also a reduction in the thermal cycle. This results in a colder FSP condition and consequently reduces the recrystallized grain size [39]. The hardness of the PZ increased with increasing the travel speed, as shown in Figure 18. It can be remarked from the hardness map that is described in blue color with a hardness value of about 29 HV (Figure 18a) at the 50 mm/min travel speed, and the color map changed to red color with hardness values around 36 HV (Figure 18d) using the Cy pin at the 300 mm/min travel speed. The correlations between microstructures and mechanical properties in terms of hardness and joint strength of the friction stir-welded aluminum alloys under different travel speeds have been studied in many works [39,40]. Increasing travel speeds can improve the mechanical properties of the friction stir welded joints through the enhancement of the material flow and plastic deformation [41,42], but extremely high travel speed can cause defects such as voids, tunnels, kissing bond, and lack of penetration due to the lack of heat input [43,44]. In contrast, extremely low travel speed promotes very high heat input, which can cause a serious softening manner in the weld zone. Lin et al. [40] studied the effect of travel speed (50–200 mm/min) on the microstructure and mechanical properties of 12-mm thick Al–Zn–Mg alloy at a constant rotation speed of 450 rpm. They found that both the average grain sizes of the shoulder-affected zone, NZ, and the widths of TMAZ decreased with the increase of travel speed.

**Figure 19:**
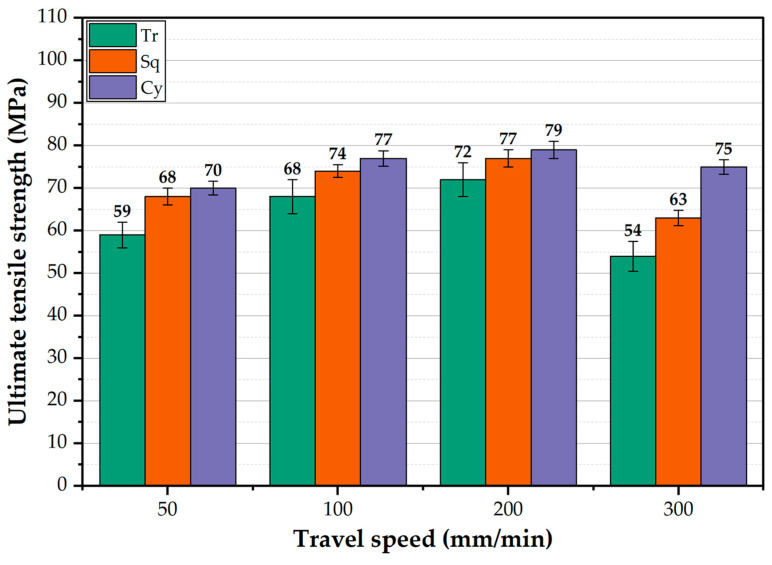
The UTS of the BT-FSPed AA1050 uses different pin geometries (Tr, Sq, and Cy) at travel speeds of 50, 100, 200, and 300 mm/min with applying a constant rotation speed of 600 rpm.

**Figure 20:**
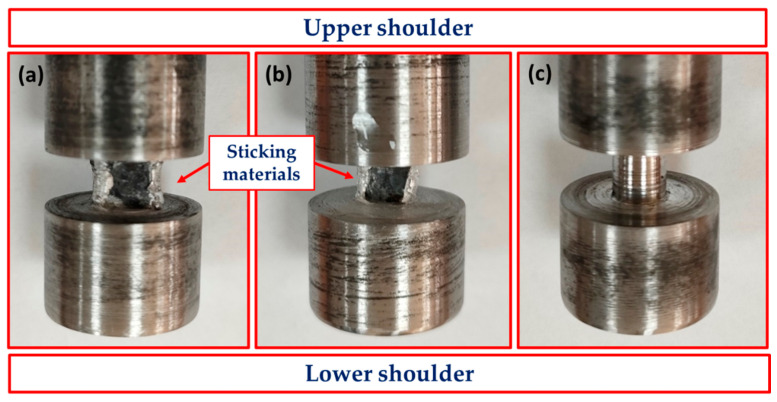
Photo images showing the sticking of plasticized aluminum of the different pin geometries of (**a**) Tr, (**b**) Sq, and (**c**) Cy used in BT-FSP of 8 mm thickness AA1050.

The ultimate tensile strength (UTS) of AA1050 BT-FSPed at different travel speeds ranging from 50 to 300 mm/min and 600 rpm rotation speed using the pins of Tr, Sq, and Cy is shown in Figure 19. For all the used pin geometries, the UTS value is higher than that of the BM (59 MPa) and increases with increasing the travel speed from 50 to 200 mm/min. This enhancement in strength compared to the BM is likely due to grain refining through dynamic recrystallization in the AA1050 PZ. Shigematsu et al. [45] produced grain refinement for both AA1050 rolled and annealed plates using friction stir processing [33]. It is reported that the grain refining in the NZ of the friction stir welded aluminum alloys ascribes to the dynamic recrystallization [46,47]. The fine grain structure increases the strength of the processed materials due to hindering the dislocation movement [48,49,50]. This trend of UTS values agrees well with that observed for the average hardness values processed at the same travel speed range (Figure 17). The highest UTS values of the processed materials were obtained at 200 mm/min travel speed at all applied pin geometries. These UTS values are 72, 77, and 79 MPa for the used pin geometries of Tr, Sq, and Cy, respectively, at the processing travel speed of 200 mm/min. The UTS of the BT-FSPed materials produced at 300 mm/min travel speed shows the lowest UTS values of 54 and 63 MPa using Tr and Sq pin geometries, respectively. This reduction in UTS values compared to AA1050 BM, and all the processed materials may ascribe to the formed tunnel defect using the Tr (Figure 13d) and Sq (Figure 14d) pin geometries. It is noticeable that the difference between UTS values produced by the Cy and Sq pin geometries is insignificant and falls within the error bar. Thus, among the applied processing parameters, it may be concluded that both pin geometries (Sq and Cy) could be recommended to achieve the highest UTS of AA1050 BT-FSPed at 200 mm/min processing travel speed and 600 rpm rotation speed. Goel et al. [48,49,50] investigated the effect of pin geometries (Cy, tapered Cy, Sq, Tr, and hexagonal) on microstructural and mechanical properties of the FSWed AA6063 using two butt joint configurations. The results showed that the Tapered and Cy tools showed the highest UTS; in contrast, the Tr pin displayed the lowest UTS. They ascribed the deterioration in strength to using Tr pin geometry due to inappropriate stirring action and insufficient material plasticization during FSW. From the point of view of design, tool life, and operating efficiency, it was found that the cylindrical tool is easier to manufacture and more efficient compared with the Tr and Sq pin geometries. Aluminum does not stick to it during the stirring process, unlike the tool with a triangle or square section. It is observed that there is aluminum stuck on the pin edges, as shown in Figure 20. Thus, machining is required from time to time during the BT-FSP to remove the stuck materials. This phenomenon is considered as an additional cost and time consumed during the processing than the Cy pin. Based on the mechanics and theory of machining principles, Mehta et al. [51] reported that during FSW, polygonal tool pins are subjected to severe stresses and, in some cases, loss of functionality because of the adhesion of plasticized material to their surfaces. Moreover, the computed stresses on the tool pins indicate that circular cross-sections will have lower stresses than the pins of polygonal cross-sections. In recent experimental work, Ahmed et al. [19] detected the adhesion of plasticized material on the pin edges of the Tr pin shape after the BT-FSW lap joint of AA1050.

**Figure 21:**
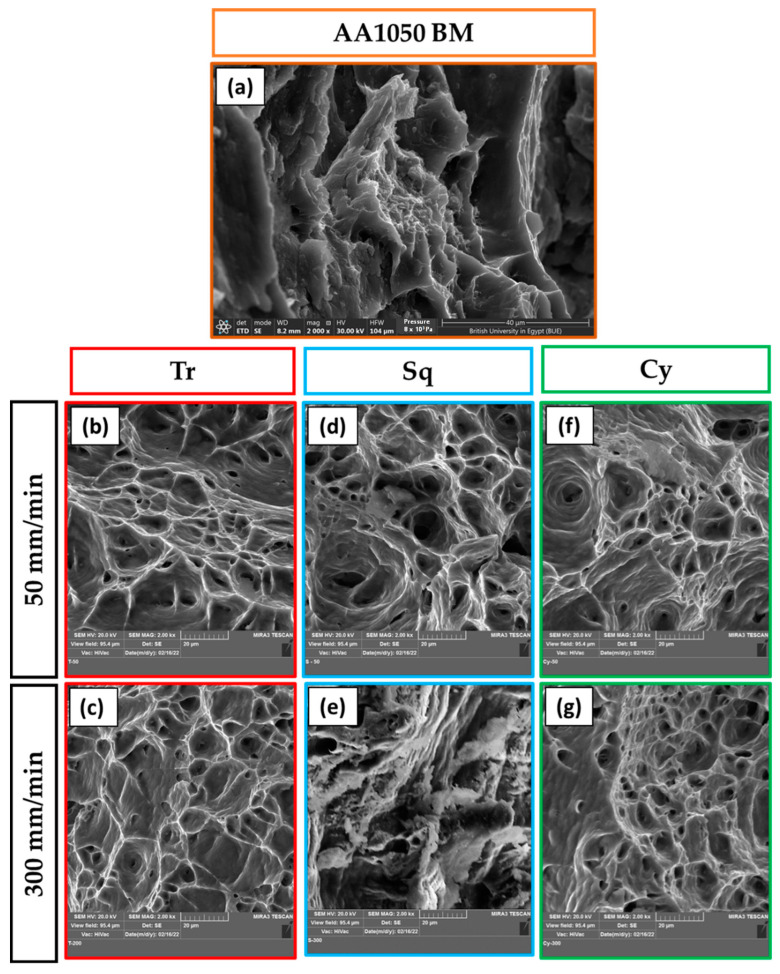
SEM images of the fracture surfaces of the failed specimens after tensile testing for AA1050 BM (**a**) and the processed materials using different pin geometries at the processing parameters of 600 rpm rotation speed and travel speeds of 50 mm/min (**b**,**d**,**f**) and 300 mm/min (**c**,**e**,**g**).

**Figure 22:**
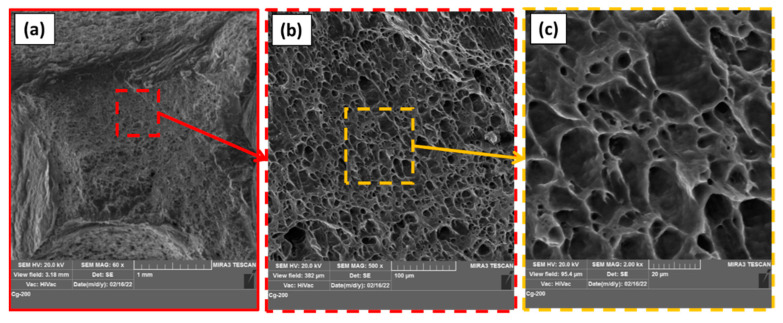
SEM images of the fracture surfaces of the BT-FSPed at 600 rpm and 200 mm/min using Cy pin geometry at different magnifications of (**a**) 60×, (**b**) 500×, and (**c**) 2000×, respectively.

Figure 21 represents the fracture surfaces of the failed specimens after tensile testing for AA1050 BM (Figure 21a) and the processed materials using different pin geometries at the processing parameters of 600 rpm rotation speed and travel speeds of 50 mm/min (Figure 21b,d,f) and 300 mm/min (Figure 21c,e,g). The fractography SEM image of AA1050 BM shows large and small dimples with tearing edges and serrations, indicating ductile fracture, as shown in Figure 21a. In general, the fracture surfaces of AA1050 processed using different pin geometries (Figure 21b–g) contain equiaxed deep and shallow dimples that are smaller in size than detected for the BM. This denotes grain refining in the PZ because of dynamic recrystallization combined with the applied processing of travel speeds and pin geometries at a constant rotation speed of 600 rpm. Furthermore, the materials processed using different pin geometries of Tr, Sq, and Cy at a lower travel speed of 50 mm/min (a higher heat input) show large and deep dimples, as given in Figure 21b,d,f, respectively, compared to those processed at the higher travel speed of 300 mm/min (a lower heat input) using Tr (Figure 21c), Sq (Figure 21e), and Cy (Figure 21g). Finally, the fracture surface of the BT-FSPed specimen at 200 mm/min travel speed and 600 rpm rotation speed using Cy pin geometry is dominated by equiaxed, uniform, and homogeneous smaller dimples with thinner edges (Figure 22a–c) compared to the AA1050 BM (Figure 21a), and all the AA1050 processed specimens (Figure 21b–g), indicating more grain refining. These features are confirmed with the highest attained mechanical properties of tensile strength and hardness.

## 4. Conclusions

BT-FSP of 8 mm thickness AA1050 was carried out at processing travel speed ranging from 50 to 300 mm/min and a constant rotation speed of 60 rpm using different pin geometries of Tr, Cy, and Sq. Based on the obtained results, it is possible to conclude the following:

1. In the BT-FSP, the travel speed and the pin geometry are two essential factors that control the temperature of the PZ. In addition, the Cy pin promotes a higher PZ temperature than other pin geometries.
2. The temperature of the advancing side is higher than the retreating side under any processing condition using the applied pin geometries.
3. The BT-FSP machine torque values increase with increasing the processing travel speed from 50 to 300 mm/min at all applied pin geometries. The highest torque value of 73 N.m was recorded using the Sq pin profile at 300 mm/min.
4. BT-FSP of AA1050 using Cy pin leads to an 8 mm full-thickness defect-free processing path at all the travel speeds. Furthermore, the processing path using the Tr and Sq obtained sound paths at 50, 100, and 200 mm/min travel speeds.
5. At 300 mm/min travel speed, Tr and Sq pins show tunnel defects which cause deterioration of the UTS.
6. Under all applied conditions, the hardness of the PZ increases with increasing travel speed. The Cy pin geometry reveals a higher hardness than the other pin geometries.
7. The AA1050 BT-FSPed using Cy pin at 200 mm/min travel speed and rotation speed of 600 rpm delivers a sound processing path with the highest ultimate tensile strength of 79 MPa with an enhancement of 33.8 % over the BM.
8. The optimized BT-FSP parameters of 8 mm thickness AA1050 to achieve the high hardness and UTS with a sound processing path are 200 mm/min travel speed and 600 rpm rotation speed using Cy pin geometry.
9. From an economic point of view, the Cy pin geometry is recommended to BT-FSP AA1050 instead of the Tr and Sq pin geometries.

## Figures and Tables

**Table 1:** The chemical composition and mechanical properties of AA1050 plates.

Chemical Composition									
Element	Cu	Zn	Mg	Mn	Cr	Ti	Si	Fe	Al
Wt. %	0.0031	0.0019	0.0030	0.0002	0.0012	0.0139	0.0889	0.257	Bal.
Mechanical Properties									
Property	UTS (MPa)			E (%)			HV		
AA1050	59 ± 2			37 ± 3			31 ± 2		

## Data Availability

Data will be available upon request through the corresponding author.
